# Dietary crocin reverses melanoma metastasis

**DOI:** 10.7555/JBR.31.20160120

**Published:** 2018-01-26

**Authors:** Hamid A Bakshi, Faruck Lukmanul Hakkim, Smitha Sam, Farideh Javid, Luay Rashan

**Affiliations:** 1. Department of Pharmacy, School of Applied Sciences, University of Huddersfield, Queensgate, Huddersfield HD13DH, United Kingdom; 2. Department of Research, Jawaharlal Nehru Cancer Hospital and Research, Idgah Hills, Bhopal 462001 MP, India; 3. Mathematics and Science Unit, College of Arts and Applied Sciences, Dhofar University, Salalah 211, Oman; 4. Frankincense Biodiversity Lab, Research center, Dhofar University, Salalah 211, Oman.

**Keywords:** dietary crocin, melanoma, lung metastasis, B16F-10, E-cadherin, MMPs, ERKs

## Abstract

*Crocus sativus* and its bioactive constituent crocin are well known for anti-tumor potential in different models. However, the efficacy of crocin on *in-vivo* melanoma metastasis is not yet reported. In this study, melanoma metastatic model was developed by tail vein injection of B16F-10 cells in to C57BL/6 mice. Metastatic mice treated with two different doses of crocin (250 and 500 µg/kg of bodyweight) for 10 days and parameters such as lung metastasis inhibition, mean survival time, lung hydroxyproline, uronic acid and hexosamine levels were analyzed after 21 days of treatment. Then blood was collected and serum gamma glutamyl transpeptidase ( g-GGT), sialic acid, tumor necrosis factor alpha (TNF-α), interleukin 10 (IL-10), IL-6, IL-2, and TIMP-1 levels were measured. Further, a lung histological examination was done in crocin treated metastatic mice. Subsequently hallmark metastatic parameters such as matrix metalloproteinases (MMPs), extracellular regulated kinase 2 (ERK2), vascular endothelial growth factor (VEGF), and K-ras gene expression were investigated in the lungs of crocin treated metastatic mice. Further, *in-vitro* adhesion, invasion and migration of B16F-10 cells were examined after 24 hours of crocin (5 and 10 µg/mL) treatment. Administration of crocin to tumor bearing C57BL/6 mice reduced the lung metastasis by 85%. Elevated levels of hydroxyproline, uronic acid, hexosamine, serum sialic acid and g-GGT in metastatic control were found to be significantly reduced in crocin treated mice. Crocin also inhibited expression of MMP-2, MMP-9, ERK-2, K-ras, and VEGF. Crocin reduced the ability of B16F-10 cells invasion (*P*<0.05), migration (*P*<0.05) and adhesion by upregulating E-cadherin expression. In conclusion, crocin elicited marked anti-metastatic potential by regulating the metastasis induced biomarkers.

## Introduction

Malignant melanoma is a potentially fatal form of skin cancer, with a strong capacity for invasion and metastasis, and high rates of recurrence and mortal-ity^[1– 2]^. The median survival following the onset of distant melanoma metastasis is just 6-9 months and the five year survival rate is<5%^[3]^. Melanoma progression and metastasis is necessarily a complex multistep process, as a cancer cell must acquire the ability to survive under anchorage independent conditions, invade through the surrounding stroma and migrate, intravasate into the vascular system; extravasate and subsequently endure a disadvantageous distant environment, adhere to the local tissue, and proliferate^[4]^.

Matrix metalloproteinases (MMPs), which are the most important factor secreted by tumor cells, stromal fibroblasts, or infiltrating inflammatory cells in the tumor microenvironment, have been strongly implicated in multiple stages of invasive and metastatic progression of tumor cells. Furthermore, MMP-9 is important for tumor angiogenesis by enhancing the availability of vascular endothelial cell growth factor (VEGF) in malignant tumors^[5]^. The interaction of VEGF with its cognate receptor VEGFR on the surface of endothelial cells promotes the recruitment and proliferation of endothelial cells *via* the activation of PI3K/Akt and Ras/Raf/MEK/ERK signaling^[6]^. In the majority of cancer patients, by the time of diagnosis of the primary tumor, metastasis to the regional lymph node and/or distant organs has occurred^[7]^. For patients with metastatic melanoma, the current therapy is based on the use of the alkylating agent dacarbazine, and some patients also receive interleukin (IL) - 2 systemically^[8]^. However, 90% of cancer deaths are caused by metastasis that are resistant to conventional therapies such as radiation and chemotherapy^[9]^. There are several drugs that are available for cancer therapy; however, not a single drug emerged as a promising agent to halt these multiple steps of melanoma metastasis.

Plants have a long history of use in the treatment of cancer^[10]^. Almost 60% of drugs approved for cancer treatment are of natural origin^[11]^. Many experimental studies and clinical trials showed that many natural plants played an important role in blocking lung metastasis from primary tumors^[12– 14]^. Many herbal drugs such as alcoholic extract of *Thuja occidentalis*^[15]^, aqueous-methanol (3:7) extract of *Boerhaavia diffusa*^[13]^, methanolic extract of *Withania somnifera* roots^[12]^, naturally occurring allyl and phenyl isothiocyanates^[16]^, curcumin^[17]^, and sulphorafane^[14]^, etc. have been reported to inhibit melanoma metastasis.

In this study we tried to explore anti-metastatic potential of *Crocus sativus* L in a melanoma model. *C. sativus* commonly known as saffron which is a dietary herb of the *Iridaceae* family. Principal components of saffron are safranal, crocin, picrocrocin and crocetin and they are pharmacologically active^[18]^. Anti-cancer and anti-tumor properties of saffron have been studied in several cancer cell lines and animal models^[19]^. It is well reported that saffron can induce apoptosis in different cancer cell lines^[20– 21]^. Our previous studies show that saffron can inhibit the growth of different cancer cells such as breast^[22]^, pancreatic^[23]^, and lung^[22]^; and it is also shown to be an active tumor remission agent in Dalton's lymphoma model^[24]^. Extract of Italian *C. sativus* has been shown to be an anti-proliferative agent in B16-F10 melanoma cell line^[25]^. Crocin is a major active component of saffron as reported by our group and elsewhere. Crocin possesses significant anti-proliferation effects on human colorectal cancer cells^[26]^. This carotenoid can induce significant alteration of gene expression profile of T24 (transitional cell carcinoma of bladder) cells. Anti-tumor effects of crocin are medicated at least in part by regulating the cell cycle controlling gene expression^[27]^. However, efficacy of crocin on *in-vivo* melanoma metastasis and its prognostic biomarkers is not yet reported. To address this issue, in this study we delineate the role of crocin on in-vivo melanoma lung metastasis.

## Materials and methods

### Plant material and extraction

Saffron stigma was powdered using mortar and pestle. Powdered materials were extracted with ethanol and it is stored in -20°C until use. Active components were identified by HPLC.

### Isolation and characterization of crocin from ***C. sativus***

Crocin was isolated from saffron as previously described^[28]^. Briefly, 500 mg saffron was washed thrice with 20 mL ethyl ether, and the residual ether was evaporated in air. It was then suspended in 15 mL of 30% methanol (v/v) in distilled water and stirred for 5 minutes at room temperature. The extract was filtered through a 0.45 mm Millipore filter. It was then diluted with 10 mmol/L phosphate buffered saline (PBS, pH= 7.4), and the concentration of crocin was adjusted to 25 mmol/L, using the coefficient €_443_ = 89,000 M^−^^1^ cm^−^^1^ reported for crocin in aqueous solution^[29]^. The crocin structure was elucidated on the basis of ^1^HNMR, ^13^CNMR, IR, and mass spectral data.

### ***In-vitro*** anti-metastatic studies

#### Cell lines and culture method

B16F-10 (CRL-6475) melanoma cells were purchased from ATCC, USA. Cells were cultured in Dulbecco's modified eagle's medium (DMEM) with 10% FBS and 1% antibiotics (penicillin/streptomycin) and maintained in humidified cell incubator at 37°C and 5% CO_2_.

#### Tumor cell adhesion assay

Tumor cell adhesion assay was carried out as described earlier^[30]^. Briefly, B16F-10 cells were seeded on to type I collagen coated wells of flat-bottomed titer plates, in the absence and presence of crocin (5 mg/mL, 10 mg/mL) and incubated at 37°C for 24 hours. After incubation, cells were washed with PBS and adherent cells were fixed, stained with Giemsa staining and counted under a microscope. Data were presented as mean±SD of triplicates of three independent experiments.

#### Collagen matrix invasion assay

Tumor cell invasion assay was carried out in modified Boyden Chamber as described earlier^[31]^. Briefly, the lower compartment of the chamber was filled with serum free DMEM and polycarbonate filter of 8 mm pore size was placed above this. Each filter was coated with 25 mL of type I collagen to form a thin continuous film on the top of the filter. B16F-10 melanoma cells (10^5^/150 mL DMEM) were added to the upper chamber and incubated at 37°C in 5% CO_2_ for 24 hours in the presence and absence of different concentrations of crocin (5 mg/mL, 10 mg/mL). After 24 hours of incubation, the cells on the lower surface of the membrane filter were fixed, stained and counted. Data were presented as percentage of invasion of triplicates of three independent experiments.

#### Tumor cell migration assay

Tumor cell migration assay was performed similar to invasion assay except that polycarbonate filters were collagen free. Crocin (5 mg/mL, 10 mg/mL) was added along with B16F-10 melanoma cells to the upper compartment of the Boyden chamber. After incubation at 37°C for 24 hours, the number of cells migrating to the lower chamber was determined using a haemocytometer. The results are expressed as percentage motility of triplicates of three independent experiments.

#### Determination of the effect of dietary crocin on expression of E-cadherin expression

The whole cell lysate was prepared from crocin (10 µg/mL) treated B16F-10 melanoma cells after 24 hours as described earlier^[32]^. Then whole cell lysate were resolved in a 10% SDS polyacrylamide gel electrophoretically and electro transferred onto a nitrocellulose membrane. The immunoblots was probed with anti-E-cadherin antibody and visualized with the NBT/BCIP chromogenic substrate and documented.

### ***In-vivo*** anti-metastatic studies

#### Animals

Six to eight week old male C57BL/6 mice were used for the study. Mice were maintained under standardized, environmental conditions (22-28°C, 60%-70% relative humidity, 12 hours dark/light cycle and water ad libitum). All the experiments were conducted under the guidelines of Institutional Animal Ethical Committee.

#### Induction of metastasis

Metastasis was induced to animals by injecting B16F-10 melanoma cells (1×10^6^ cells/animal) *via* the lateral tail vein^[33]^.

#### Drug preparation

Crocin was dissolved in minimum volume of ethanol and re-suspended in 1% gum acacia and was given to animals intraperitoneally (i.p) at a concentration of 250 µg and 500 mg/kg body weight.

#### Determination of in-vivo anti-metastatic potential of crocin

After induction of metastasis, the mice were divided in to four groups (*n* = 12). Group I animals were kept as normal control and group II as metastatic tumor bearing control receive saline intraperitoneally. Group III and IV received 250 µg and 500 mg/kg body weight, respectively, of crocin intraperitoneally for 10 days consecutively from the day of tumor induction. After the treatment period, six animals from each group were sacrificed and then blood was collected by heart puncture and the serum was separated. Further, lungs were excised and thoroughly washed in PBS and peripheral lung nodules were counted and used to estimate various biochemical parameters such as hydroxyproline, hexosamine, and uronic acid. Serum g-glutamyl transpeptidase ( g-GT) and sialic acid were estimated by ELISA. The remaining six animals in all of the groups were observed for survival. Histopathological analysis was carried out by fixing both whole lungs, treated and untreated control animals in formaldehyde (10%) and then dehydrated using gradient alcohol and embedded in paraffin wax. Sections (4 mm) were stained with hematoxylin and eosin.

#### Determination of the effect of dietary crocin on cytokine and TIMP-1 production in metastatic tumor bearing animals

Metastasis was induced in 4 groups of C57BL/6 mice (*n* = 12). Group I was normal control and Group II was metastatic control. Group II and III animals had received crocin at 250 µg and 500 mg/kg body weight, respectively, continuously for ten days. Six animals from each group were sacrificed on day 7 and 21 after treatment. Then serum cytokines such as IL-10, IL-6, TNF-α, IL-2 and TIMP-1 were measured using respective ELISA kits by following the manufacturer's instruction.

#### Gene expression profile of MMP-2, MMP-9, ERK-2, VEGF, and K-ras

The animals were sacrificed on day 21 after treatment. Then lungs were excised and RNA was extracted using guanidium thiocynate and cDNA was synthesized as described elsewhere. PCR was performed using specific primers of MMP-2, MMP-9, VEGF, Erk-2, and *K*-ras. PCR products were resolved by agarose gel electrophoresis and visualized using ethidium bromide dye.

#### Statistical evaluation

*In-vitro* data presented as mean±SD of triplicates of three independent experiments. *In-vivo* data were presented as mean±SD of two triplicates. Experimental data was evaluated by Students' *t*-test and Graph PAD In stat software, Kyplot. Significant differences between each set of data were considered at the confidence level of *P*<0.05 and *P*<0.001.

## Results

### Inhibition of lung metastasis by crocin

**Fig.1:**
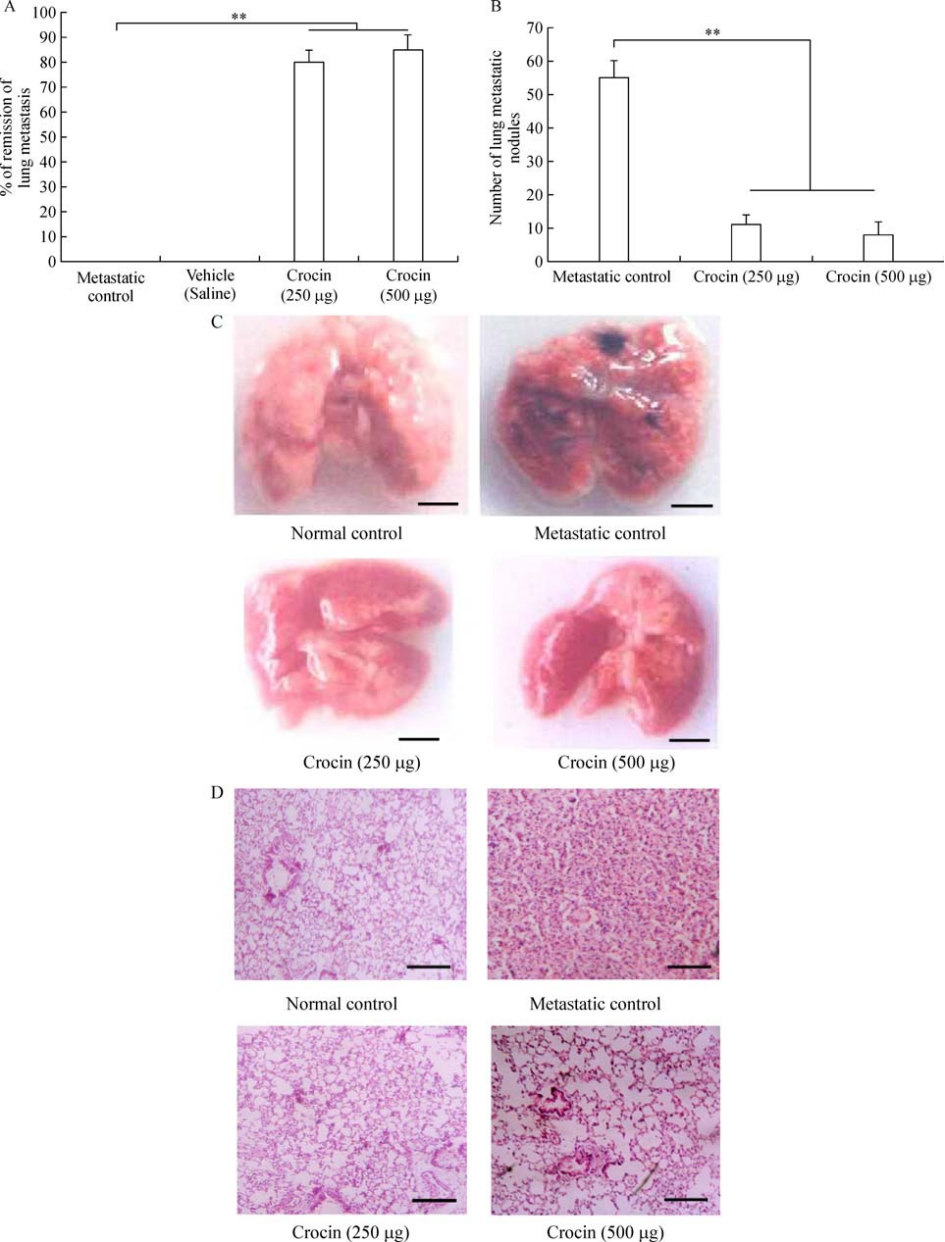
Effect of dietary crocin on lung metastasis.

A significant reduction in the number of pulmonary metastatic colonies of B16F-10 melanoma cells were observed in crocin treated mice compared to metastatic tumor bearing control. Administration of crocin 250 and 500 µg/kg of bodyweight reduced percent of lung metastasis by 80% and 85% respectively in a dose dependent manner (*P*<0.001) (***Fig. 1A***). Further morphology and histopathological data clearly showed significant reduction of lung metastasis in crocin treated mice compared to untreated metastatic control (***Fig. 1B, C***). These data strongly support the anti-metastatic activity of dietary crocin.

### Mean survival time and body weight of crocin treated mice

**Fig.2:**
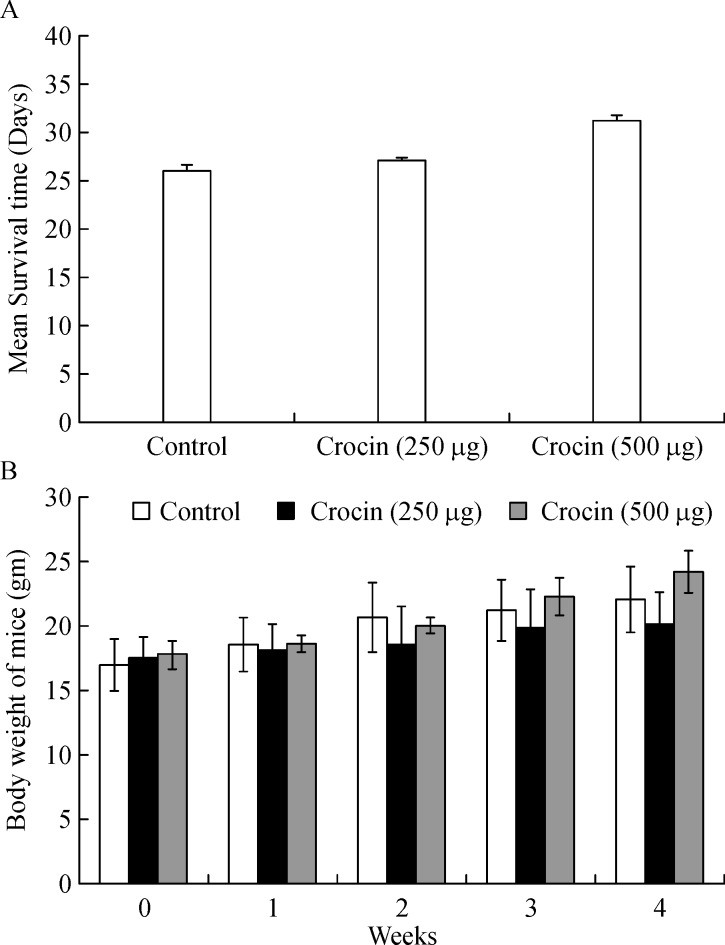
Effect of dietary crocin on mean survival time and body weight.

Mean survival time of crocin (250 and 500 µg/kg of body weight) treated mice was extended up to 27.1 and 31 days respectively compared to untreated metastasis control mice where the survival ended after 26 days of the experimental period (***Fig. 2A***). Furthermore, body weight was measured in normal mice fed with crocin (250 and 500 µg/kg of body weight) for four weeks. We found no significant difference in bodyweight of crocin treated mice compared to control mice (***Fig. 2B***). This data reveals that crocin can be tolerated by mice without eliciting toxicity.

### Efficacy of crocin on reduction of lung hydroxyproline, uronic acid and hexosamine content

**Fig.3:**
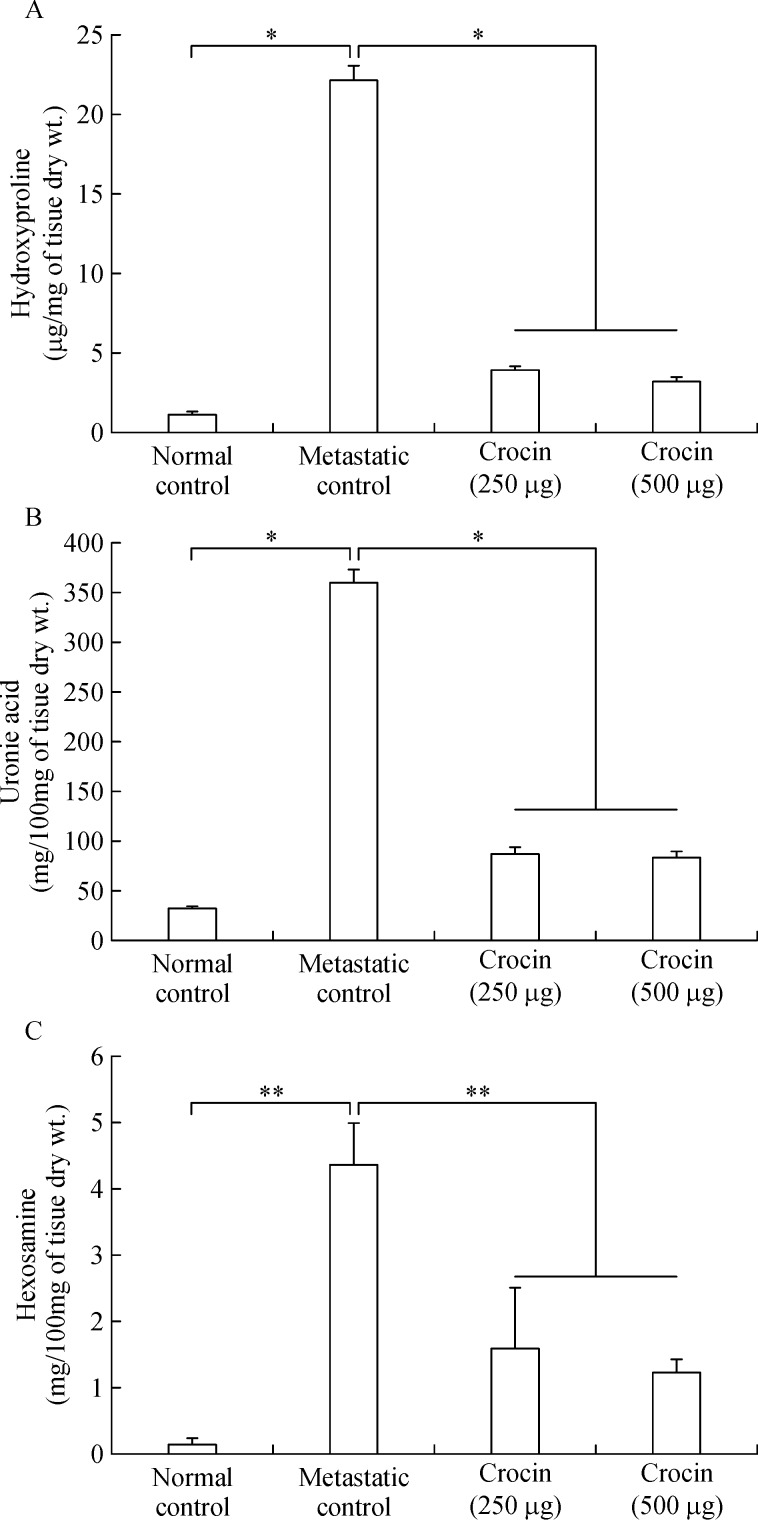
Effect of dietary crocin on the lung biochemical parameters.

Elevated levels of hydroxyproline (22.17±0.93 mg/mg of tissue dry weight), uronic acid (360.58±13.04 mg/100 mg tissue dry weight), and hexosamine (4.36±0.63 mg/100 mg tissue dry weight) was observed in metastatic control mice. Moreover, 250 and 500 mg/kg of body weight crocin treatment reduced their levels significantly and brought back near to normal control ***(Fig. 3A, B, C).***

### Role of crocin on serum γ- GGT and sialic acid

**Fig.4:**
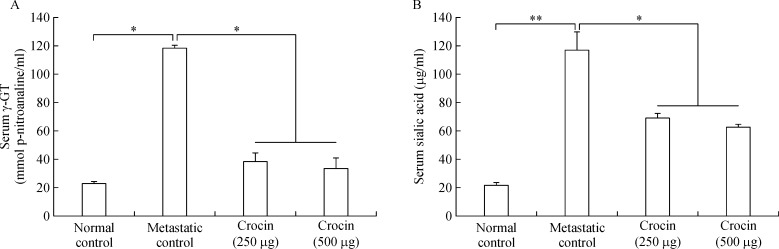
Effect of dietary crocin on the serum γ-GT and sialic acid levels in metastasis bearing animals.

As serum sialic acid and g-GGT are lung metastatic biomarkers in this study, we measured their levels in crocin treated metastatic mice. Both sialic acid (21.78±1.98 mg/mL) and g-GGT (118.11±5.83 nmol p-nitroaniline/mL) levels increased significantly in metastatic mice compared to normal control. Crocin treatment reduced sialic acid and g-GGT levels significantly at the end of experimental period (***Fig. 4 A, B***).

### ***In-vitro*** adhesion, invasion and migration of B16F-10 cells upon crocin treatment

**Fig.5:**
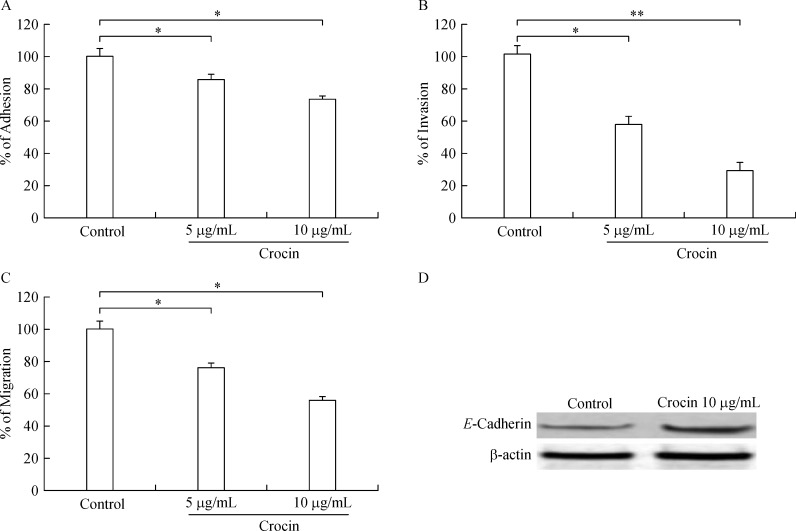
Effect of dietary crocin on adhesion, invasion, migration and E-cadherin expression.

B16F-10 cells treated with crocin (5 and 10 µg/mL) for 24 hours showed dose-dependent decline in adhesion (85.5% and 73.5%, respectively), invasion (57.2% and 29.2%, respectively), and migration (76.03% and 55.87%, respectively) (***Fig. 5A, B, C***). Further, high expression of *E*-cadherin was observed in crocin (10 µg/mL) treated B16F-10 cells ***(Fig. 5D).***

### Activation of TIMP1 and deactivation of MMP (2 and 9), VEGF, ERK2, and K-ras by crocin

**Fig.6:**
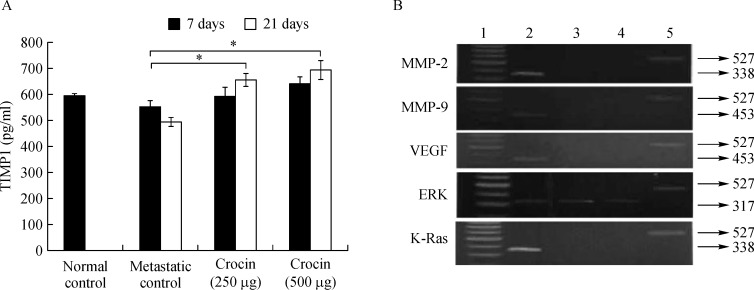
Effect of dietary crocin on the TIMP-1 level, MMP-2 and MMP-9, VEGF, ERK, and K-Ras expression in metastasis bearing animals.

As MMP2 and MMP9 facilitates the detachment of tumor cells from primary tumor site and VEGF promotes dissemination *via* ERK2 and Ras, TIMP1 is directly involved in inhibition of MMP's and halt metastatic process. To determine the role of crocin on these signaling molecules in this study, we measured the serum TIMP1 level and expression of MMP's, ERK2 and Ras in crocin treated mice. Serum TIMP-1 level in untreated metastatic tumor bearing control mice was 553.96±21.41 pg/mL and increased with treatment of crocin after 7 days (250 µg/ kg of body weight (591.71±71.33 pg/mL), 500 µg/ kg of body weight (640.20±24.09 pg/mL) and 21 days (250 µg/ kg of body weight (654.29±23.67 pg/mL), 500 µg/ kg of body weight (693.8±35.8 pg/mL) (***Fig. 6A***). Further expression of MMP-2, MMP-9, VEGF, Erk-2 and Ras (***Fig. 6B***) were considerably reduced by crocin treatment in comparison to metastatic control.

### Efficacy of dietary crocin on serum TNF-α, IL-6, IL-2, and IL-10

**Fig.7:**
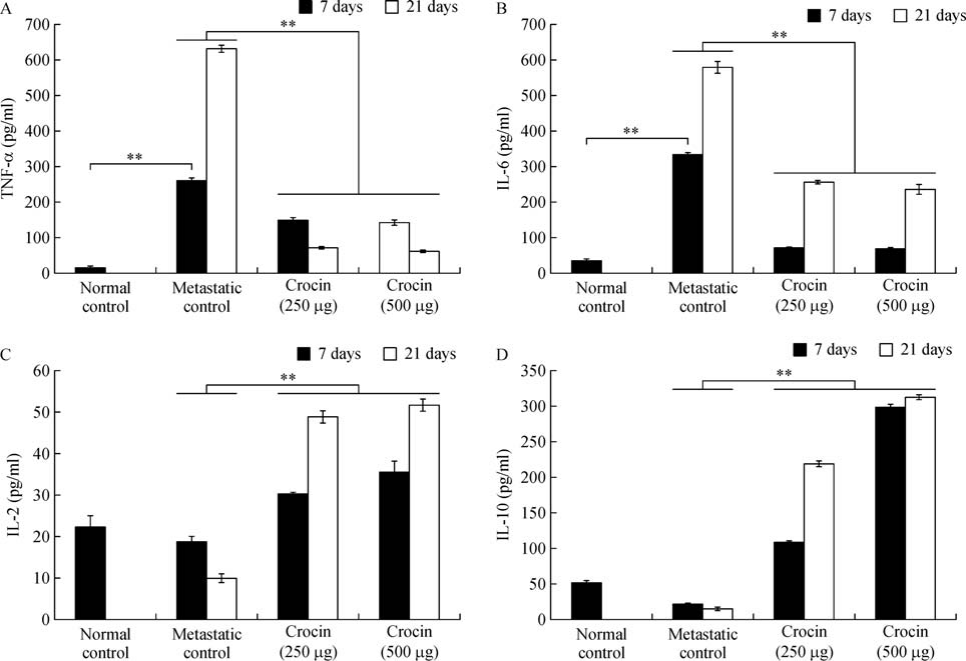
Effect of dietary crocin on serum TNF-α, IL-6, IL-2 and IL-10 in in metastatic bearing animals.

TNF-α (day 7: 262.12±8.42 pg/mL; day 21: 630.80±9.54 pg/mL) and IL-6 (day 7: 335.08±3.65 pg/mL; day 21: 580.28±16.6 pg/mL) were significantly elevated in metastatic mice compared to normal control. Administration of crocin at 250 and 500 µg/kg of body weight reduced TNF-α (day 7: 153.3±5.53 pg/mL and 145.45±5.3 pg/mL, respectively; day 21: 74.78±3.42 pg/mL and 65.43±2.33 pg/mL, respectively), IL-6 (day 7: 72.07±1.66 pg/mL and 68.77±3.77 pg/mL, respectively; day 21: 257.43±4.39 pg/mL and 235.89±12.2 pg/mL, respectively) levels significantly (*P*<0.001) (***Fig. 7A, B***). In contrast, IL-2 level was reduced on day 7 (18.67±1.3 pg/mL) and slightly elevated on day 21 (630.8±9.54 pg/mL) in metastatic mice compared to control. Similarly, IL-10 level also declined on day 7 (21.23±0.95 pg/mL) and enhanced on day 21 (630.8±9.54 pg/mL) in metastatic mice compare to normal control. Treatment with crocin at 250 and 500 µg/ kg of body weight enhanced IL-2 (day 7: 30.2±0.32 pg/mL and 35.46±2.65 pg/mL, respectively; day 21: 48.71±1.26 pg/mL and 51.51±1.31 pg/mL respectively), IL-10 (day 7: 108±2.51 pg/mL and 297±5.27 pg/mL, respectively; day 21: 218±4.18 pg/mL and 312±2.78 pg/mL, respectively) levels significantly (*P*<0.001) (***Fig. 7C, D***).

## Discussion

Advanced metastatic melanoma is highly recalcitrant to treatment due to massive spreading and its aggressive nature. Targeted immunotherapy using a cocktail of monoclonal antibodies elicits severe post treatment allergic reactions. In this scenario, recent scientific efforts have focused on the potential roles of traditional herbs as an alternative and complementary medication for cancer treatment. Extensive research on dietary herbs over the past two decades found that some herbs inhibit different types of tumor growth *in-vivo* and halt *in-vitro* cancer cell proliferation. Many plant derived bioactive constituents, including paclitaxel (from *Taxus brevifolia*), camptothecin (from *Camptotheca acuminata*), podophyllotoxin (from *Podophyllum emodi*), and vinblastine (from *Catharanthus roseus*), have been developed as potential sources of anticancer agents^[34]^. *Crocus sativus* and its active constituent crocin is tumor inhibitory agent in a variety of *in-vivo* tumor models^[19]^. Crocin is the major constituent of *C. sativus* shown to be an effective anti-tumor agent^[22– 23]^. However, efficacy of crocin on melanoma metastasis is not yet reported. It is well known that B16F-10 melanoma cells are highly metastatic and form tumor nodules in the parenchyma of the lungs when administered through the tail vein^[33]^. Therefore, in this study, we tested the efficacy of crocin on metastatic melanoma model generated by tail vein injection of B16-F10 cells.

Administration of crocin at 250 and 500 µg/ kg of body weight showed significant inhibition of lung metastasis at about 80% and 85% respectively after 21 days of the experimental period (***Fig. 1***). Tumor nodules are metastatic colonies of B16F-10 melanoma cells formed in the lungs, and they initiate lung fibrosis and collagen deposition. Tumor nodule inhibition by drugs correlated with an increase in the life span of the metastatic tumor bearing animals. In our study, mean survival of metastatic mice fed with crocin (500 µg/kg of body weight) extended up to 31 days compare to control (***Fig. 2***). As per the WHO, dietary herbs and their constituents are highly tolerable by the human system compared to synthetic products. Our previous study showed that 300 mg/kg of body weight of *C. sativus* extract administration to normal mice is safe and there was no adverse histopathologic differences observed in the major organs^[35]^. The extent of lung fibrosis during metastasis correlated with lung collagen hydroxyproline content because during lung fibrosis, collagen is deposited massively in the alveolus of the lungs. Fifteen to thirty percent of collagen is hydroxyproline^[36]^, which results in the reduction of pulmonary function. High content of uronic acid results in accumulation of hydroxyproline in lung metastasis. Tumor cells produce uronic acid as a result of oxidation of aldoses of sugar derivatives and leads to the formation of esterified form of glucuronic acid. Conversion of prohydroxyproline to hydroxyproline by prolyl hydroxylase facilitated by the presence of glucuronic acid. In addition glucuronic acid also activates the fiber formation during lung fibrosis^[34]^. Followed by uronic acid, hexosamine is a significant sugar derivative present in the tumor cells. It has an important role in the synthesis of n-acetylneuraminic acid (sialic acid), which is a component of glycolipids present on the surface of tumor cells, thus promoting growth and dissemination^[36]^. In our study, the level of these molecules was enhanced in the lung tissue of metastatic control. On treatment with crocin, the level of uronic acid, hydroxyproline, and hexosamines brought back significantly and this observation indicates reduced metastatic burden (***Fig. 3A, B, C***). Above mentioned biochemical parameters are interconnected to each other to facilitate lung metastasis and it is directly correlated with the degree of lung metastasis. Overall, our data reveals that levels of hydroxyproline, uronic acid and hexamine in the lungs of metastatic mice treated with crocin (250 and 500 µg/kg of body weight) directly correlated with our lung metastasis remission data (***Fig. 1A***) where we did not observe drastic differences between the doses. However, the molecular mechanism responsible for lack of dose-dependency is unclear.

Rapidly growing tumors need cysteine for intracellular reduced glutathione (GSH) synthesis in order to obtain energy and to sustain growth and subsequent dissemination. g-glutamyl transpeptidase (GGT) cleaves GSH releasing g-glutamyl amino acids and cysteinylg lycine, which is further cleaved by membrane-bound dipeptidases into cysteine and glycine^[37]^. Further free g-glutamyl-amino acids, cysteine, and glycine entering the cell serve as GSH precursors^[38]^. Hence, GGT expression facilitates tumor burden and metastatic growth. In our study, we found that serum GGT levels were enhanced in metastatic control but crocin treatment reduced its level significantly (***Fig. 4A***) and it is evidenced from histopathological studies where the metastatic nodules have disappeared after crocin treatment. Sialic acids that are aberrantly expressed on cancer cells appear to facilitate several steps of the metastatic cascade^[39]^. In the current study, we found that elevated serum sialic acid level in metastatic control. High sialic acid expression favors metastasis by facilitating cancer cell detachment and protection from detachment-induced apoptosis (anoikis), enhancing migration and tissue invasion by increasing integrin interactions with the ECM, enabling interactions with endothelial cells to extravasate from the blood stream and form metastases^[40]^. Therefore, inhibiting sialic acid expression could be of crucial importance to halt metastatic cascade and subsequent colonization at distant organs. Our result shows that crocin treatment reduces the level of serum sialic acid significantly at the end of the experimental period (***Fig. 4B***). Molecular mechanism of crocin on reducing sialic acid expression in metastatic mice is unclear; however, it could be due to inhibitory effect of crocin on the metastasis initiation cascade such as adhesion, invasion and migration. The invasion of tumor cells into adjacent tissues, a crucial event in metastasis, involves cell–cell and cell–ECM interactions^[41]^. These interactions involve a number of adhesive molecules on the cell surface, which have been described in detail^[42]^. Drugs that can inhibit the adhesion of the cells to the ECM may have anti-metastatic potential. To address this issue in this study, we evaluated the potential of crocin on *in-vitro* adhesion, invasion and migration of B16-F10 cells. We found that there was dose-dependent decline in the adhesion of B16-F10 cells with collagen matrix, invasion and migration after crocin treatment (***Fig. 5A, B, C***). Loss of *E*-cadherin facilitates detachment and migration of tumor cells^[43]^. In our study, crocin treatment restores the expression of *E*-cadherin compares to metastatic control (***Fig. 5D***).

Matrix metalloproteinases (MMPs), MMP2 and MMP9 in particular, have been regarded as major molecules that assist tumor cells by cleaving several ECM components and it paves the way for detachment and dissemination during metastasis^[44]^. Tissue inhibitors of metalloproteinases (TIMPs) act as natural inhibitors of MMPs by tightly binding the MMP in a 1:1 stoichiometric ratio and are associated with normal and pathological ECM turnover^[45]^. TIMP inhibits MMP activity, thereby suppressing tumor invasion and metastasis. This phenomenon led us to determine the efficacy of crocin on MMP 2, 9 and TIMP1. Level of TIMP1 reduced in metastatic control but crocin treatment enhanced their levels after 7 and 21 days of the experimental period (***Fig. 6A***). Furthermore, crocin administration inhibited expression of MMP 2 and 9 in lung tissue of treated mice (***Fig. 6B***). Our results are in agreement with previous studies where enhanced TIMP expression can decrease metastasis and inhibit angiogenesis in experimental mouse model^[46]^. Angiogenesis is the hallmark of metastasis and vascular endothelial growth factor (VEGF) is highly expressed in aggressive melanoma cell lines and that melanoma patients with higher VEGF concentrations have a higher rate of relapse^[47]^. VEGF is one of the external stimuli to induce the expression of MMP's and it would facilitate migration. Inhibition of tumor growth has been achieved in different melanoma xenograft models through the use of a number of anti-VEGF strategies^[48]^. As we observed crocin treatment inhibited expression of MMP's, we speculate that crocin could be anti-VEGF component. Our data shows that mice treated with 250 and 500 µg/ kg of body weight of crocin completely lost the expression of VEGF compared to metastatic control (***Fig. 6B***). However, the molecular mechanism of crocin on VEGF remains unclear. Survival, proliferation and invasive responses of tumor cells have been shown to be mediated by VEGF through Erk (1/2) pathways^[49]^. Furthermore, the interaction of VEGF on its cognate receptor VEGFR of endothelial cells requires functional activation of ERK (1/2) pathway, which operates downstream of Ras, is often upregulated in tumors and represents an attractive target for anticancer therapy^[50]^. To identify the role of crocin on Ras/ERK signaling in melanoma lung metastasis in this study, we determine the gene expression pattern of Ras/ERK in crocin treated mice. Crocin (250 and 500 µg/kg of body weight) fed mice was associated with inhibition of Ras and dose dependent decline of ERK2 expression ***(Fig. 6B).*** Our data suggest that crocin inhibits angiogenesis by suppressing VEGF *via* downregulating ERK2 and its downstream Ras ***(Fig. 6B).*** Similarly, thujone, a monoterpene that inhibits melanoma lung metastasis by downregulating ERK (1/2) and its downstream Ras^[50]^.

Cytokines play a pivotal role in triggering metastasis. TNF-α shown to be an important cytokine mediator of cancer metastasis in murine models^[51]^. Ocvirk *et al*. found that concentrations of TNF-α were significantly higher in metastatic melanoma patients^[52]^ and Cubillos *et al*. suggested that the reduction in the number of metastasis may be related to the effect of blocking TNF activity in melanoma cells^[53]^. Similarly, in our study, we found that the serum TNF-α level increased significantly in metastatic control mice but crocin treatment reduced its level after 7 and 21 days of the experimental period ***(Fig. 7A).*** Furthermore, in this study, we measured serum interleukin (IL) 2, 6, and 10 levels in metastatic and crocin treated mice. IL-6 is secreted by malignant melanoma cells, and the serum level is associated with advanced stages of the disease. Noticeably, serum IL-6 level was found to be significantly high in advanced melanoma patients^[54]^. In our study, we found that crocin treatment significantly reduced serum IL-6 level compared to metastatic control ***(Fig. 7B).*** This implies that immune modulating ability of crocin in melanoma metastasis. IL-2 has been used for more than two decades in the therapy of metastatic melanoma which, in some of treated patients, resulted in induction of long-lasting remission^[55]^. Forced expression of IL-10 in human melanoma cells did not enhance tumor growth and metastatic potential in nude mice, but rather significantly inhibited their tumorigenicity and metastatic capabilities^[56]^. In the current study, both IL-2 and IL-10 levels were enhanced significantly by crocin treatment at the end of the experimental period (***Fig. 7C, D***). IL-10 has been shown to inhibit the production of IL-1, IL-6, IL-8, TNF-α^[57]^ and forced expression of IL-10 suppressed VEGF and MMP-9, secreted by tumor-associated macrophages in melanoma model^[56]^. Our results are in agreement with this report since crocin treatment elevated the levels of IL-10 and IL-2.

In conclusion, our experimental data has clearly demonstrated that dietary crocin strongly inhibits lung metastasis of B16F-10 melanoma cells *in-vivo*. Furthermore, crocin suppresses *in-vitro* adhesion, invasion, and migration by upregulating E-cadherin. However, extensive preclinical study is required to transform dietary crocin as a cancer therapeutic agent for melanoma patients.
